# Incidence of atrial functional tricuspid regurgitation and its correlation with tricuspid valvular deformation in patients with persistent atrial fibrillation

**DOI:** 10.3389/fcvm.2022.1023732

**Published:** 2022-12-20

**Authors:** Yuko Yamamoto, Masao Daimon, Koki Nakanishi, Tomoko Nakao, Megumi Hirokawa, Jumpei Ishiwata, Hiroyuki Kiriyama, Yuriko Yoshida, Kentaro Iwama, Kazutoshi Hirose, Yasuhiro Mukai, Norifumi Takeda, Yutaka Yatomi, Issei Komuro

**Affiliations:** ^1^Department of Cardiovascular Medicine, The University of Tokyo Hospital, Tokyo, Japan; ^2^Department of Clinical Laboratory, The University of Tokyo Hospital, Tokyo, Japan

**Keywords:** atrial fibrillation, tethering height, tricuspid annular dilation, tricuspid regurgitation, valvular heart disease

## Abstract

**Background:**

With the growing prevalence of atrial fibrillation (AF), concomitant atrial functional tricuspid regurgitation (FTR) is increasing. In this study, we aimed to elucidate the incidence of significant atrial FTR and its association with tricuspid valvular (TV) deformation in patients with persistent AF.

**Methods:**

We retrospectively enrolled 344 patients (73.0 ± 9.3 years, 95 female) with persistent AF who underwent 2-dimensional echocardiography. We excluded patients with left-sided heart disease, pulmonary hypertension treated with pulmonary vasodilators, and congenital heart disease. We defined significant TR as having TR ≥ moderate; and tricuspid annulus (TA) diameter, tethering height, and area were measured in all patients.

**Results:**

Among the study population, 80 (23.3%) patients had significant TR. TA diameter, tethering height, and area were significantly greater in the significant TR group (all *p* < 0.001). In multivariable analysis, TA diameter was independently associated with significant TR (odds ratio 1.1 per mm, *p* = 0.03), whereas TV tethering height was not. Receiver operating characteristic curve for significant TR exhibited the best predictive value of TA diameter indexed for body surface area [23 mm/m^2^; area under the curve (AUC) = 0.87] compared with absolute TA diameter (39 mm; AUC = 0.74) and TA diameter indexed for height (0.22 mm/cm; AUC = 0.80).

**Conclusion:**

Approximately 25% of patients with persistent AF had significant TR. The BSA-corrected TA diameter was strongly associated with significant TR, which might be helpful for predicting the development of significant TR and considering its therapeutic strategy in patients with persistent AF.

## Introduction

The number of patients with atrial fibrillation (AF) is growing worldwide with aging populations (1). The estimated global burden of AF in 2010 was 33.5 million, and since then the number has been rapidly increasing (2). This epidemiological transition has drawn attention to concomitant atrial functional tricuspid regurgitation (FTR), which plays a critical role in the development of heart failure in AF patients (3, 4). Recent studies suggest that atrial FTR has different mechanisms from conventional ventricular FTR, which is mainly attributed to right ventricular (RV) enlargement and tricuspid valve (TV) leaflet tethering secondary to left heart disease (4–6). A possible important etiology for atrial FTR is tricuspid annular (TA) dilation (4–8). However, these previous studies (4–8) included left heart disease, congenital heart disease, or pulmonary disease, which may cause pulmonary hypertension and morphological changes of the right heart. Thus, the incidence of atrial FTR and its correlation with TV deformation in patients with isolated persistent AF has not been extensively studied, which may limit the therapeutic intervention. This present study sought to analyze isolated persistent AF patients excluding those with left heart disease, pulmonary hypertension, or congenital heart disease that may cause TV tethering and TA dilation. Furthermore, whereas concomitant TV surgery may be indicated for patients with TA dilation (> 40 mm or 21 mm/m^2^) (9–11) at left-sided valve surgery, the rationality of indexing TA diameter for body size in atrial FTR has not been fully investigated. This study therefore aimed to investigate: (i) the incidence of significant atrial FTR; (ii) the association of TV deformation parameters, including TA diameter, TV tethering height, and tethering area with atrial FTR; (iii) the reliability of indexation of TA diameter for the detection of atrial FTR in patients with persistent AF and without left heart disease, pulmonary hypertension, or congenital heart disease.

## Materials and methods

### Study population

**FIGURE 1:**
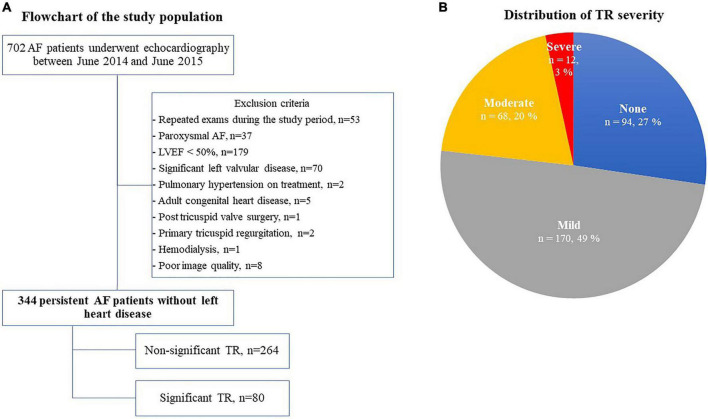
Flow chart of the study population **(A)** and the distribution of TR severity **(B)**. AF, atrial fibrillation; LVEF, left ventricular ejection fraction; and TR, tricuspid regurgitation.

We retrospectively enrolled consecutive persistent AF patients who underwent echocardiography at the University of Tokyo Hospital from June 2014 to June 2015. All clinical data were collected through a review of electronic medical records. Exclusion criteria were as follows: paroxysmal AF, significant left valvular disease (≥ moderate aortic/mitral regurgitation or stenosis), left ventricular (LV) dysfunction [LV ejection fraction (EF) < 50%], pulmonary hypertension on treatment with pulmonary vasodilators, congenital heart disease, post TV surgery, primary TR, hemodialysis, peritoneal dialysis, post heart transplantation, wearing a ventricular assist device, repeated examinations, and inadequate echocardiographic images (Figure 1A). We confirmed the duration of AF based on the electrocardiogram or on the medical records, and identified the patients with persistent AF. Persistent AF was defined as AF that lasted for more than 7 days according to the guidelines (12). All patients were divided into the following two groups: a non-significant TR group with mild or less TR, and a significant TR group with moderate or greater TR. We defined moderate TR as significant TR, because a recent study showed that individuals with moderate TR had a 2.4-fold risk of cardiovascular death when compared with those with no/trivial TR (13). The study was approved by the ethics committee of the University of Tokyo Hospital (IRB number 3825).

### Echocardiographic assessment

**FIGURE 2:**
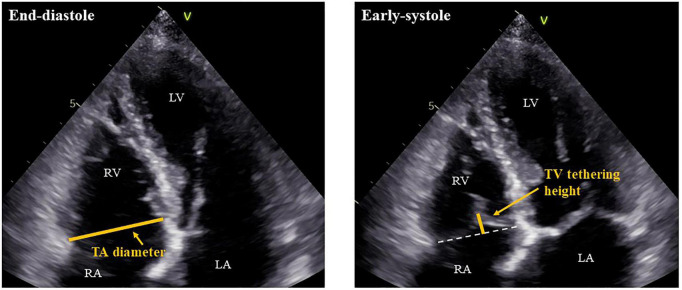
Measurements of TA diameter and TV tethering height. RV, right ventricle; RA, right atrium; LV, left ventricle; LA, left atrium; TA, tricuspid annulus; and TV, tricuspid valve.

Patients in AF rhythm underwent 2-dimensional and Doppler echocardiography by experienced operators in accordance with the guidelines of the American Society of Echocardiography (14). Our echocardiography laboratory is maintained according to the guidelines of the Japanese Society of Echocardiography (15). LV volume was measured by disk summation in apical four-chamber and two-chamber views, and LVEF was evaluated by Simpson’s biplane method. When images of a two-chamber view were not appropriate for evaluation, we used only a four-chamber view to obtain LVEF. LV mass was calculated by the following formula: LV mass (g) = 0.8 × {1.04 × [(IVST + LV EDD + PWT)^3^ – (LVEDD)^3^]} + 0.6, where IVST is interventricular septum thickness, LV EDD is LV end-diastolic diameter, and PWT is posterior wall thickness (14). LV mass was divided by body surface area (BSA) to create LV mass index. We used pulse-wave Doppler echocardiography in the apical four-chamber view to assess early diastolic transmitral flow velocity (E). The early diastolic peak tissue Doppler imaging velocity (e’) was measured at the septal and lateral annulus sites in the apical four-chamber view and was averaged. The ratio of the E-wave to the mean e’ velocity (E/e’) was calculated to evaluate LV filling pressure (16). Left atrial (LA) volume was calculated from the apical four-chamber and two-chamber views using Simpson’s biplane method. When that was not possible, LA volume was measured only from the four-chamber view. LA volume was then indexed to BSA. RV and RA areas as well as TA diameter, TV tethering height, and TV tethering area were obtained from the RV-focused apical four-chamber view (17). TA diameter was measured at end diastole, while TV tethering height and area were measured at early systole (Figure 2). For assessing the reproducibility of echocardiographic measurements of TA diameter, TV tethering height, and TV tethering area, two independent observers repeated the measurements subsequently in 10 cases, and intraclass correlation coefficients (ICC) were calculated.

RV contractility was evaluated by fractional area change (FAC) (13). FAC was calculated from the RV-focused apical four-chamber view by the following formula: FAC (%) = [(RVEDA – RVESA)/RVEDA] × 100, where RVEDA is RV end-diastolic area and RVESA is RV end-systolic area. When an ideal RV-focused apical four-chamber view was not available, they were measured in the standard apical four-chamber view. The peak systolic velocity of the tricuspid annulus (s’) was also measured using tissue Doppler imaging. Semi-quantitative assessment of TR was performed according to the guidelines of the American Society of Echocardiography and graded as none, mild, moderate, or severe (18). TR pressure gradient (TRPG) was calculated from the continuous-wave Doppler TR velocity by the simplified Bernoulli equation. For all measurements, one beat with an average R-R interval and/or velocity was carefully selected.

### Statistical analysis

Continuous data (variables) were expressed as mean ± standard deviation (SD) or median (interquartile range). Categorical variables were presented as number of patients (percentage). Differences between patients with and without significant TR were compared with Student’s *t*-test or the Wilcoxon rank-sum test as appropriate. For categorical variables, Pearson’s chi-square test was performed. Univariable and multivariable logistic regression analyses were used to identify the determinants of significant TR, and odds ratios (ORs) along with 95% confidence intervals (CI) reported. Those with a *p*-value < 0.05 on univariable analysis were selected as independent variables for multivariable analysis. To clarify the best indexation of TV parameters for significant atrial FTR, receiver operating characteristic (ROC) curves were constructed to evaluate and compare the area under the curve (AUC). Two-sided *p*-values < 0.05 were considered to indicate statistical significance. JMP Pro 15.0 (SAS Institute, NC, USA) was used for all statistical analyses.

## Results

### Baseline characteristics

We screened 702 AF patients who underwent echocardiography between June 2014 and June 2015, 358 of whom were excluded according to our exclusion criteria (Figure 1A). Of the remaining 344 patients with persistent AF, 80 patients (23.3%) had significant TR (severe TR in 12 patients and moderate TR in 68 patients) (Figure 1B). Clinical characteristics are shown in Table 1. The significant TR group was older, had a greater prevalence of females and lower BMI. Of note, the prevalence of females in the significant TR group was double that in the non-significant TR group (45.0% vs. 22.4%). While there were no significant differences in symptoms or other comorbidities between the 2 groups, the number of patients receiving diuretics was greater in the significant TR group. In the laboratory tests, the significant TR group had lower hemoglobin, and higher bilirubin and BNP levels (all *p* < 0.05).

**TABLE 1:** Patient characteristics.

	Overall, *n* = 344	Non-significant TR group, *n* = 264	Significant TR group, *n* = 80	*P*-value
Age, years	73.0 ± 9.3	71.5 ± 9.3	77.9 ± 7.7	<0.00001
Females, *n* (%)	95/344 (27.6%)	59/264 (22.3%)	36/80 (45.0%)	<0.0001
BMI, kg/m^2^	23.3 ± 3.5	23.9 ± 3.5	21.5 ± 2.7	<0.0001
sBP, mmHg	126.9 ± 18.0	127.0 ± 18.0	126.3 ± 18.2	0.79
dBP, mmHg	74.1 ± 13.3	74.9 ± 13.2	71.3 ± 13.1	0.04
HR, bpm	78.4 ± 16.1	78.8 ± 15.9	77.2 ± 16.5	0.31
NYHA				0.36
I	298/344 (86.6%)	232/264 (87.9%)	66/80 (82.5%)	
II	31/344 (9.0%)	21/264 (8.0%)	10/80 (12.5%)	
III	12/344 (3.5%)	8/264 (3.0%)	4/80 (5.0%)	
IV	3/344 (0.9%)	3/264 (1.1%)	0/80 (0.0%)	
**Comorbidities**				
Diabetes mellitus, *n* (%)	87/344 (25.3%)	74/264 (28.0%)	13/80 (16.3%)	0.03
Hypertension, *n* (%)	214/344 (62.2%)	165/264 (62.5%)	49/80 (61.3%)	0.84
Dyslipidemia, *n* (%)	132/344 (38.4%)	107/264 (40.5%)	25/80 (31.3%)	0.13
History of smoking, *n* (%)	135/233 (57.9%)	106/176 (60.2%)	29/57 (50.9%)	0.21
COPD, *n* (%)	13/344 (3.8%)	9/264 (3.4%)	4/80 (5.0%)	0.51
CKD, *n* (%)	193/344 (56.1%)	153/264 (58.0%)	40/80 (50.0%)	0.21
CAD, *n* (%)	53/343 (15.5%)	43/263 (16.3%)	10/80 (12.5%)	0.40
Stroke, *n* (%)	47/343 (13.7%)	36/263 (13.7%)	11/80 (13.8%)	0.99
Past history of heart failure, *n* (%)	109/344 (31.7%)	77/264 (29.2%)	32/80 (40.0%)	0.07
**Past cardiac surgery**				
Aortic valve, *n* (%)	17/344 (4.9%)	15/264 (5.7%)	2/80 (2.5%)	0.25
Mitral valve, *n* (%)	31/344 (9.0%)	22/264 (8.3%)	9/80 (11.3%)	0.42
CABG, *n* (%)	12/344 (3.5%)	9/264 (3.4%)	3/80 (3.8%)	0.88
**Medications**				
Beta blocker, *n* (%)	167/344 (48.5%)	127/264 (48.1%)	40/80 (50.0%)	0.77
CCB, *n* (%)	140/344 (40.7%)	104/264 (39.4%)	36/80 (45.0%)	0.37
RAS-I, *n* (%)	179/344 (52.0%)	141/264 (53.4%)	38/80 (47.5%)	0.35
Diuretic, *n* (%)	120/344 (34.9%)	83/264 (31.4%)	37/80 (46.3%)	0.01
AAD, *n* (%)	98/344 (28.5%)	72/264 (27.3%)	26/80 (32.5%)	0.36
**Laboratory data**				
Hemoglobin, g/dL	13.6 (12.3–15)	14 (12.5–15.4)	13.1 (11.8–13.8)	<0.0001
eGFR, mL/min/1.73 m^2^	58.4 (46.7–69)	57.8 (47.9–69)	56 (44.8–69.1)	0.72
Total bilirubin, mg/dL	0.8 (0.6–1.1)	0.7 (0.6–1)	1 (0.7–1.3)	0.01
Sodium, mEq/L	140 (139–142)	141 (139–142)	140 (138–141)	0.06
BNP, pg/mL	143.85 (89–216.8)	132.75 (84.4–196.3)	180.5 (109.5–239.9)	0.01

Data are expressed as mean ± *SD*. Laboratory data are expressed as median (25–75% percentile). TR, tricuspid regurgitation; BMI, body mass index; sBP, systolic blood pressure; dBP, diastolic blood pressure; HR, heart rate; NYHA, New York Heart Association; COPD, chronic obstructive pulmonary disease; CKD, chronic kidney disease; CAD, coronary artery disease; CABG, coronary artery bypass graft; CCB, calcium channel blocker; RAS-I, renin-angiotensin system inhibitor; AAD, antiarrhythmic drug; eGFR, estimated glomerular filtration rate; BNP, brain natriuretic peptide.

### Echocardiographic parameters

Table 2 shows the echocardiographic parameters of the study population. In the entire population, LV and RV systolic function were preserved, whereas LA and RA dilation were notable. The significant TR group had a relatively smaller LV volume and LV mass index, while they exhibited a larger RV area and RA area index, and higher TRPG. As for TV geometry, the significant TR group had significantly greater TA diameter, TV tethering height, and tethering area (all *p* < 0.001). The intra- and inter-observer variabilities of TA diameter, TV tethering height, and TV tethering area, were 4.9 ± 3.9% (ICC: 0.949) and 5.6 ± 3.2% (ICC: 0.949), 9.2 ± 3.7% (ICC: 0.988), and 11.0 ± 3.7% (ICC: 0.951), and 8.8 ± 3.5% (ICC: 0.958) and 8.4 ± 7.0% (ICC: 0.995), respectively.

**TABLE 2:** Echocardiography parameters stratified by presence or absence of significant TR.

	Overall, *n* = 344	Non-significant TR, *n* = 264	Significant TR, *n* = 80	*P*-value
LV EDV index, ml/m^2^	56.7 ± 17.0	58.3 ± 17.5	51.4 ± 13.7	0.0013
LV ESV index, ml/m^2^	22.2 ± 8.6	23.0 ± 9.0	19.4 ± 6.6	0.0007
LV mass index, g/m^2^	89.8 ± 24.3	91.8 ± 24.1	83.3 ± 23.8	0.0033
LVEF, %	61.2 ± 7.1	60.9 ± 7.1	62.3 ± 7.1	0.09
E/e’	12.2 ± 6.7	12.4 ± 7.1	11.7 ± 5.1	0.90
LAV index, ml/m^2^	64.9 ± 36.6	62.1 ± 28.5	74.0 ± 54.5	0.27
RV EDA index, cm^2^/m^2^	10.5 ± 3.1	10.0 ± 2.5	12.1 ± 4.1	<0.0001
RVESA index, cm^2^/m^2^	6.0 ± 1.9	5.7 ± 1.7	6.7 ± 2.4	0.0030
RV FAC, %	42.8 ± 10.2	42.2 ± 10.5	44.8 ± 9.2	0.04
RV s’, cm/s	10.1 ± 2.4	9.9 ± 2.4	10.6 ± 2.3	0.13
RA area index, cm^2^/m^2^	15.5 ± 5.0	14.2 ± 3.6	19.6 ± 6.4	<0.0001
TRPG, mmHg	25.5 ± 8.2	24.1 ± 7.7	30.2 ± 8.4	<0.0001
TA diameter, mm	35.2 ± 5.6	34.0 ± 4.8	39.2 ± 6.5	<0.0001
TV tethering height, mm	5.4 ± 2.3	5.1 ± 2.1	6.5 ± 2.6	<0.0001
TV tethering area, cm^2^	1.1 ± 0.7	1.0 ± 0.6	1.5 ± 0.9	<0.0001

Data are expressed as mean ± *SD*. LV, left ventricular; EDV, end diastole volume; ESV, end systole volume; LVEF, left ventricular ejection fraction; LAV, left atrial volume; RV, right ventricle; EDA, end diastole area; ESA, end systole area; FAC, fractional area change; RA, right atrium; TRPG, tricuspid regurgitation pressure gradient; TA, tricuspid annular; TV, tricuspid valve.

### Determinants of significant TR

The results of univariable and multivariable logistic regression analyses for the significant TR group are shown in Table 3. Among the TV deformation variables, both larger TA diameter index and TV tethering heights were correlated with significant TR in the univariable model (both *p* < 0.0001). In multivariable analysis, TA diameter detected significant FTR independent of age, BMI, and left heart size, as well as RV and RA size, whereas TV tethering height did not.

**TABLE 3:** Univariable and multivariable logistic regression analyses for significant TR.

	Univariable odds ratio (95%CI)	*P*-value	Multivariable odds ratio (95%CI)	*P*-value
Age, years	1.1 (1.06–1.13)	<0.0001	1.1 (1.04–1.19)	0.0006
Female	2.8 (1.68–4.82)	<0.0001	1.3 (0.44–3.89)	0.63
BMI, kg/m^2^	0.8 (0.73–0.87)	<0.0001	0.8 (0.65–0.91)	0.0009
sBP, mmHg	0.998 (0.98–1.01)	0.78		
dBP, mmHg	0.98 (0.959–0.999)	0.03	0.8 (0.98–1.06)	0.45
HR, bpm	0.993 (0.98–1.09)	0.41		
NYHA ≥ III	1.2 (0.38–3.91)	0.75		
**Comorbidities**				
Diabetes mellitus	0.5 (0.26–0.96)	0.03	0.7 (0.27–2.04)	0.56
Hypertension	0.9 (0.57–1.589)	0.84		
Dyslipidemia	0.7 (0.39–1.13)	0.13		
History of smoking	0.7 (0.38–1.25)	0.22		
COPD	1.5 (0.45–4.98)	0.53		
CKD	0.7 (0.44–1.20)	0.21		
CAD	0.7 (0.35–1.53)	0.39		
Stroke	1.005 (0.49–2.08)	0.99		
History of heart failure	1.6 (0.96–2.72)	0.07		
**Past cardiac surgery**				
Aortic valve	0.4 (0.10–1.90)	0.22		
Mitral valve	1.4 (0.61–3.16)	0.44		
CABG	1.1 (0.29–4.18)	0.89		
**Medications**				
B eta-blocker	1.1 (0.65–1.78)	0.77		
CCB	1.3 (0.76–2.09)	0.37		
RAS-I	0.8 (0.48–1.30)	0.35		
Diuretic	1.9 (1.1–3.1)	0.02	0.7 (0.29–1.87)	0.52
AAD	1.3 (0.7–2.2)	0.37		
**Laboratory data**				
Hemoglobin, g/dL	0.8 (0.72–0.90)	0.0002	0.8 (0.67–1.06)	0.14
eGFR, mL/min	1.003 (0.99–1.02)	0.62		
Total bilirubin, mg/dL	0.99 (0.94–1.05)	0.81		
Sodium, mEq/L	0.9 (0.79–1.01)	0.06		
Log_10_BNP, pg/mL	2.5 (1.03–6.06)	0.04	1.8 (0.41–8.15)	0.43
**Echocardiography parameters**				
LV EDV index, ml/m^2^	0.97 (0.954–0.989)	0.0007	0.9 (0.90–0.98)	0.0011
LV mass index, g/m^3^	0.98 (0.973–0.996)	0.0049	0.99 (0.96–1.01)	0.19
LV EF, %	1.03 (0.993–1.065)	0.12		
E/e’	0.98 (0.94–1.03)	0.41		
LAV index, ml/m^2^	1.008 (1.00–1.02)	0.02	1.01 (1.00–1.02)	0.048
RV EDA index, cm^2^/m^2^	1.2 (1.14–1.36)	<0.0001	1.3 (1.03–1.56)	0.02
RV FAC, %	1.03 (1.00–1.05)	0.04	1.02 (0.98–1.07)	0.34
RV s’, cm	1.1 (0.99–1.25)	0.08		
RA area index, cm^2^/m^2^	1.3 (1.20–1.38)	<0.0001	1.3 (1.15–1.52)	<0.0001
TA diameter, mm	1.2 (1.13–1.26)	<0.0001	1.1 (1.01–1.26)	0.03
TV tethering height, mm	1.3 (1.16–1.46)	<0.0001	1.2 (0.95–1.52)	0.13

CI, confidence interval; BMI, body mass index; sBP, systolic blood pressure; dBP, diastolic blood pressure; HR, heart rate; NYHA, New York Heart Association; COPD, chronic obstructive pulmonary disease; CKD, chronic kidney disease; CAD, coronary artery disease, CABG, coronary artery bypass graft; CCB, calcium channel blocker; RAS-I, renin-angiotensin system inhibitor; AAD, antiarrhythmic drug; eGFR, estimated glomerular filtration rate; BNP, brain natriuretic peptide; LV, left ventricular; EDV, end diastole volume; LVEF, left ventricular ejection fraction; LAV, left atrial volume; RV, right ventricle; EDA, end diastole area; FAC, fractional area change; RA, right atrium; TA, tricuspid annular; TV, tricuspid valve.

### Indexation of TA diameter

**FIGURE 3:**
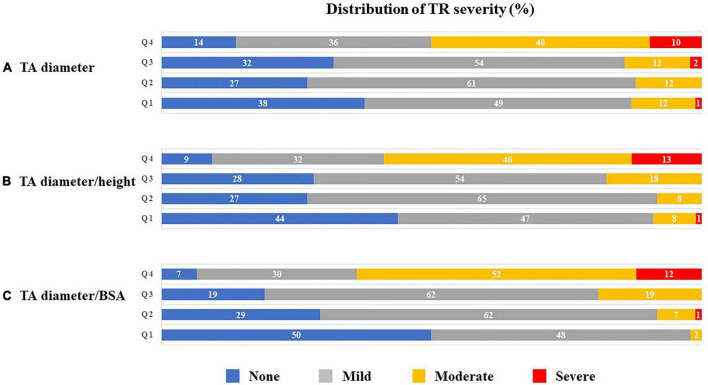
Distribution (%) of TR severity according to quartiles of TA diameter **(A)**, TA diameter/height **(B)** and TA diameter/BSA **(C)**. BSA, body surface area; TA, tricuspid annulus; and TR, tricuspid regurgitation.

**FIGURE 4:**
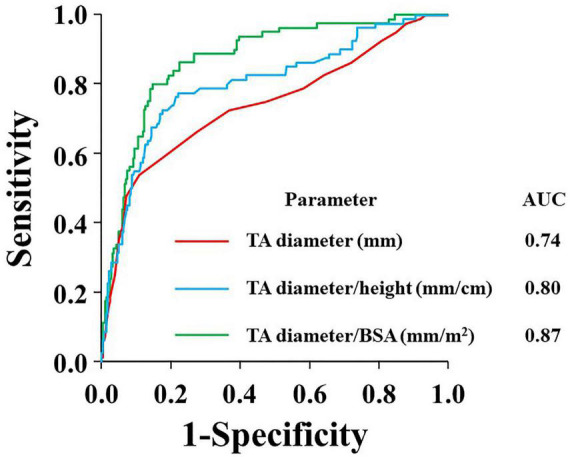
ROC curves for the prediction of significant TR according to absolute TA diameter (red), TA diameter/height (blue), and TA diameter/BSA (green). AUC, area under the curve; BSA, body surface area; TA, tricuspid annulus; and TR, tricuspid regurgitation.

Figure 3 presents the distributions of TR severity in each quartile according to the TA diameter, corrected TA diameter by body height or BSA, respectively. Next, we performed ROC analysis to evaluate the best indexation of TA diameter for the detection of significant atrial FTR (Figure 4). Indexed TA diameter for BSA exhibited the best detection value with a cutoff value of 23 mm/m^2^ (sensitivity = 80.0%, specificity = 85.4, AUC = 0.87, respectively) compared with the direct measure of TA diameter with a cutoff of 39 mm (sensitivity = 53.8%, specificity = 89.0%, AUC = 0.74, respectively) and TA diameter indexed for body height with 0.22 cm/m^2^ (sensitivity = 77.5%, specificity = 77.7%, AUC = 0.80, respectively) (all *p* < 0.001).

## Discussion

The major findings of this study were as follows: (1) Among persistent AF patients free from left heart disease, 23% had significant TR; (2) TA diameter, but not tethering height, was independently associated with significant TR; (3) BSA-indexed TA diameter showed better detection value for significant TR, with a cutoff of 23 mm/m^2^, compared with an absolute value of 39 mm.

### Incidence of significant TR in patients with persistent AF

In this cohort, nearly a quarter of persistent AF patients free from left heart disease or pulmonary hypertension had significant TR. Recent studies have reported the incidence of significant TR in AF patients. Zhou et al. showed that approximately one in three lone AF patients had significant TR (19). Abe et al. found 15% AF patients with normal EF had significant TR (3). More recently, Prapan et al. demonstrated that 21% patients had significant TR in 300 AF (paroxysmal; 35% and persistent; 65%) patients with preserved LVEF (20). Our observation is consistent with these studies, and we also strictly excluded other etiologies causing TR, which may help to identify the inherent association between AF and FTR.

### The role of TA dilation in the pathophysiology of atrial FTR

Because significant TR accompanied by AF carries significant and independent prognostic impact, unraveling the mechanism of atrial FTR is of crucial importance. Limited data are available on the association between TV deformation and atrial FTR, and the pathophysiological mechanism has not been completely explained (4); several reports have presented that TA dilation but not TV tethering was a significant determinant of atrial FTR (21, 22). On the other hand, Zhao et al. showed that TV tethering height was significantly greater in patients with significant atrial FTR, while TA diameter was not (23). This could be partially explained by the difference in study population. Our study demonstrated that both tethering height and TA diameter were significantly larger in the significant TR group in the univariable model, although only TA diameter detected significant TR in the multivariable analysis. Our observations suggest that TA dilation plays a more important role in the pathophysiological mechanism of atrial FTR compared with tricuspid leaflet tethering in persistent AF patients free from left heart disease. Of note, significant FTR was independently associated with both TA diameter and RA area, respectively, as shown in Table 3. Whereas RA enlargement might be an important determinant of TA dilation, TA dilation is presumably caused by a multifactorial mechanism including RV remodeling, or LV and LA geometrical abnormalities. As previously reported, extensive RV remodeling caused by higher severity of atrial functional TR may lead to both TA dilation and TV tethering (6). Possible hypotheses could be that even if the original pathophysiological condition was atrial functional TR, it might develop ventricular functional TR-like characteristics as it progresses. We also found that small LV size and larger LA volume were associated with significant TR, suggesting that subclinical LV diastolic dysfunction might be involved in the association (24).

### Usefulness of indexation of TA diameter

Our results suggest that TA diameter > 39 mm or TA diameter/BSA > 23 mm/m^2^ would be reasonable cutoffs for significant TR in persistent AF patients. Current guidelines recommend concomitant prophylactic TV surgery at the time of left-sided heart surgery for mild or moderate secondary TR in the presence TA dilation (i.e., TA diameter ≥ 40 mm or TA diameter/BSA > 21 mm/m) (8–10); these cut-off values are similar to those obtained in the present study. However, only a few studies have tested the validity of these cutoff values (25–27) and none of the current guidelines suggested the optimal cut-off value of TA diameter for atrial FTR. Furthermore, we examined whether indexation of TA diameter carries better detection value for significant TR, and found that BSA-indexed TA diameter had the best detection value. These findings might support the concept of indexation for TA diameter. Indeed, Yajima et al. identified TA diameter/BSA as an independent detector of significant TR development after aortic valve surgery (28).

### Body mass index and significant TR

In this study, smaller BMI was found to be independently associated with significant TR (Table 3). Recently, Dietz et al. (29) reported that in patients with significant TR, overweight and obese patients demonstrated more RV remodeling and severe TR compared with patients with normal weight. They presumed that obesity might affect RV structure by a multifactorial mechanism of increased RV afterload, increased circulating blood volume, metabolic and neuroendocrine influences, and direct obesity-related myocardial effects (30, 31). However, our results were inconsistent with their findings. In our study, malnutrition or liver dysfunction induced by significant TR may be associated with a smaller BMI. However, further studies are needed to clarify the association between BMI and significant TR.

### Clinical implications

Considering the prognostic impact of significant TR and its association with TA diameter, careful follow-up should be undertaken in persistent AF patients with TA dilation. Furthermore, patients with dilated TA may benefit from certain modifications in surgical or catheter procedure techniques. These concepts, however, require testing in prospective, large, controlled trials.

### Limitations

Because this was a single-center, observational, retrospective, cross-sectional study, selection bias cannot be ruled out and causality cannot be established. For exploring the predictive value of TA dilation for significant TR, longitudinal studies are needed with larger study populations as Nishimura et al. reported (32). In this study, we have a limited number of patients to follow since a number of them were followed by other hospitals, making it difficult to reach definitive conclusions. We understand the importance of studies including prognostic and follow-up echocardiographic data and will conduct further studies to address this issue. Furthermore, although we excluded patients with paroxysmal AF, the severity of AF burden was not uniformly available. Although we excluded patients with pulmonary hypertension on treatment with pulmonary vasodilators, and averaged TRPG was not significantly high (25.5 ± 8.2 mmHg), mild pulmonary hypertension not requiring pulmonary vasodilators or LV diastolic function might be included in our study. RV or TV geometrical abnormalities caused by these pathophysiologies might affect our results. This issue needs to be addressed in a further study with a more strictly controlled population. RV and TV geometry is technically difficult to assess accurately with 2-dimensional echocardiography due to its anatomical 3-dimensional complexity, and linear measurements of RV and TV have limited reliability in assessing these geometries. Three-dimensional echocardiography now offers an accurate evaluation of RV shape (33). Although relatively high feasibility and reproducibility of TV annulus diameter using 2-dimensional echocardiography has been reported when compared with 3-dimensional echocardiography (34), further study using 3-dimentional echocardiography is needed.

## Conclusion

Significant TR was observed in approximately 25% of patients with AF. TA dilation, but not tethering height, was independently associated with the presence of significant TR. The BSA-indexed TA diameter might have greater detection value for significant TR than absolute diameter. The BSA-indexed TA diameter, which was well-associated with significant TR, might be helpful for predicting the development of significant TR and deciding its therapeutic strategy in patients with persistent AF.

## Data availability statement

The original contributions presented in this study are included in the article/supplementary material, further inquiries can be directed to the corresponding author.

## Ethics statement

The studies involving human participants were reviewed and approved by the Ethics Committee of the University of Tokyo Hospital (IRB number: 3825). Written informed consent for participation was not required for this study in accordance with the national legislation and the institutional requirements.

## Author contributions

YYam: data curation, validation, formal analysis, and writing of the original draft. MD: conceptualization, methodology, investigation, writing—review and editing, and supervision. KN: writing—review and editing. TN, MH, JI, HK, YYo, KI, KH, YM, and NT: data curation and writing—review and editing. YYat: review, editing, and supervision. IK: writing—review and editing and supervision. All authors contributed to the article and approved the submitted version.
